# Malignant pleural mesothelioma showing rare morphology indistinguishable from myxofibrosarcoma concomitant with *EGFR*-mutated lung adenocarcinoma: A case report

**DOI:** 10.1016/j.ijscr.2021.106237

**Published:** 2021-07-27

**Authors:** Hiroko Onagi, Takuo Hayashi, Tsuyoshi Saito, Satsuki Kishikawa, Kazuya Takamochi, Kenji Suzuki

**Affiliations:** a Department of Human Pathology, Juntendo University Graduate School of Medicine, Bunkyo-ku, Tokyo 113-8421, Japan; b Department of General Thoracic Surgery, Juntendo University School of Medicine, Bunkyo-ku, Tokyo 113-8421, Japan

**Keywords:** Biphasic subtype, Homozygous deletions of p16, Malignant mesothelioma, Myxofibrosarcoma

## Abstract

**Introduction and importance:**

Primary tumors of the pleura are rare, with malignant mesothelioma being the most common of these neoplasms. Pathological diagnosis of sarcomatoid mesothelioma can be more challenging than that of epithelioid malignant mesothelioma because of its similarities with true sarcomas and restricted or inconsistent expression of mesothelial markers in immunohistochemistry analysis.

**Presentation of case:**

Here, we present an unusual case of malignant pleural mesothelioma concomitant with lung adenocarcinoma in a 72-year-old Japanese man, a smoker with no family history of cancer and asbestos exposure. Malignant pleural mesothelioma is composed of epithelial and spindle-shaped cells. Spindle-shaped cells with scant eosinophilic cytoplasm and hyperchromatic nuclei proliferated in abundant myxoid stroma containing thin-walled blood vessels, mimicking myxofibrosarcoma. The loss of BAP1 (BRCA1-associated protein 1) expression, as assessed by immunohistochemistry, and homozygous deletions of *CDKN2A*, detected using fluorescence *in situ* hybridization (FISH), were observed in both components. Targeted sequencing revealed that lung adenocarcinoma harbored *EGFR* mutations, whereas no mutations were detected in either component of biphasic mesothelioma.

**Discussion:**

Although alcian blue-stained mucins were detected in biphasic mesothelioma subsets, the clinicopathological significance of myxoid stroma in biphasic and sarcomatoid mesothelioma remains largely unknown.

**Conclusion:**

Our case presented a unique morphology mimicking myxofibrosarcoma in a sarcomatoid component of biphasic mesothelioma; therefore, it raises a question on the clinicopathological significance of myxoid stroma in sarcomatous areas of biphasic and sarcomatoid mesothelioma.

## Highlights

• A case of concomitant pleural mesothelioma and lung adenocarcinoma.
• Biphasic mesothelioma had areas mimicking myxofibrosarcoma.
• The loss of BAP1 expression was assessed by immunohistochemistry.
• Homozygous deletions of *CDKN2A* was detected by FISH.
• Lung adenocarcinoma harbored *EGFR* mutation.

## 1 Introduction

Primary tumors of the pleura are rare, with malignant mesothelioma being the most common. The 2021 World Health Organization classification defines four main histological subtypes of malignant mesothelioma: epithelioid, sarcomatoid, biphasic, and desmoplastic [1]. Pathological diagnosis of sarcomatoid mesothelioma can be more challenging than that of epithelioid malignant mesothelioma because of its similarities with true sarcomas and restricted or inconsistent expression of mesothelial markers in immunohistochemistry analysis [2]. Among sarcomatoid mesotheliomas, “conventional” sarcomatoid mesothelioma with no special subtype, which resembles fibrosarcoma or undifferentiated sarcoma, previously known as malignant fibrous histiocytoma, shows the most common histology (44%), followed by sarcomatoid mesothelioma with desmoplastic features (34%) [2]. However, it is unclear how often the myxofibrosarcoma, formerly known as the myxoid variant of malignant fibrous histiocytoma, is seen in malignant pleural mesothelioma. Furthermore, sarcomatoid mesotheliomas may exhibit leiomyosarcoma-like features [3], with the occurrence of heterologous elements such as osteosarcoma or chondrosarcoma [4]. This variation in histological appearance could be confused with soft tissue tumors, including fibrosarcoma, synovial sarcoma, leiomyosarcoma, osteosarcoma, chondrosarcoma, and undifferentiated sarcoma.

Here, we describe an unusual case of biphasic mesothelioma of the pleura with areas mimicking myxofibrosarcoma concomitant with *EGFR*-mutated lung adenocarcinoma in a 72-year-old Japanese man. The work has been reported in line with the SCARE 2020 criteria [5].

## 2 Case presentation

**Fig. 1:**
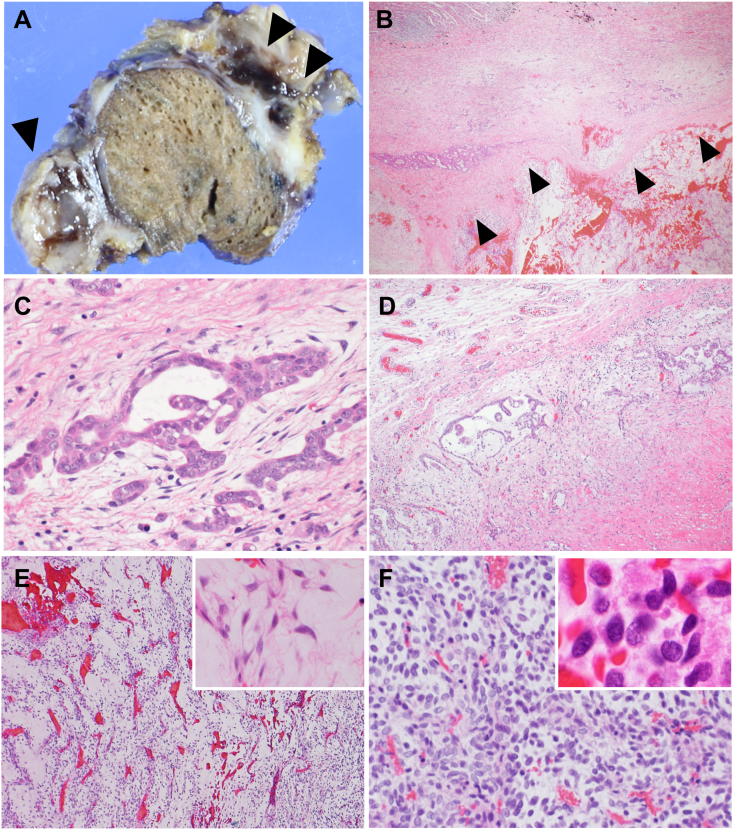
Pathological findings of biphasic malignant mesothelioma. (A) Macroscopically, black-colored myxoid nodules were identified in the visceral pleura (arrows). (B) Both epithelioid and sarcomatoid areas juxtaposed each other. Intralesional fibrous septum existed between two components (arrows). (C) Epithelial cells with ovoid nuclei formed tubular structure. (D) A gradual transition between two components. (E) Tumor cells proliferated in myxoid stroma containing thin-walled blood vessels (inset: tumor cells demonstrated spindle-shaped cells). (F) Spindle-shaped cells had hyperchromatic nuclei (inset: oil immersion view of tumor cells, original magnification x1000).

A 72-year-old Japanese man, who was a smoker (53 pack-years), presented to our hospital with a chief complaint of cough. His medical history included diabetes mellitus and dyslipidemia. He had no family history of cancer or asbestos exposure. A computed tomography (CT) scan of his chest revealed a 15 mm-sized nodule in the right upper lung (S3b) with nodules in the visceral pleura of the upper lobe of the right lung. No other abnormalities were observed. He subsequently underwent wedge resection. On macroscopic examination, a 15 × 7 mm-sized nodule in the lung parenchyma was identified, and the visceral pleura was thickened with hemorrhagic and myxoid nodules (Fig. 1A). Microscopic analysis revealed that the tumor in the visceral pleura was composed of two different cell components: epithelial and spindle-shaped cells. Each component accounted for more than 10% of the tumor. Epithelial cells with ovoid nuclei formed a tubular structure. Spindle-shaped cells with scant eosinophilic cytoplasm and hyperchromatic nuclei proliferated in myxoid stroma containing thin-walled blood vessels, mimicking myxofibrosarcoma. Intralesional fibrous septum existed between the two components; however, there was a gradual transition between the two components (Fig. 1B-F). Necrosis and scattered mitoses (8 mitoses/10 high-power fields) were detected. Neither multinucleated giant cells nor osseous or chondroid elements were observed in the sarcomatous component. Asbestos bodies were not detected.

**Fig. 2:**
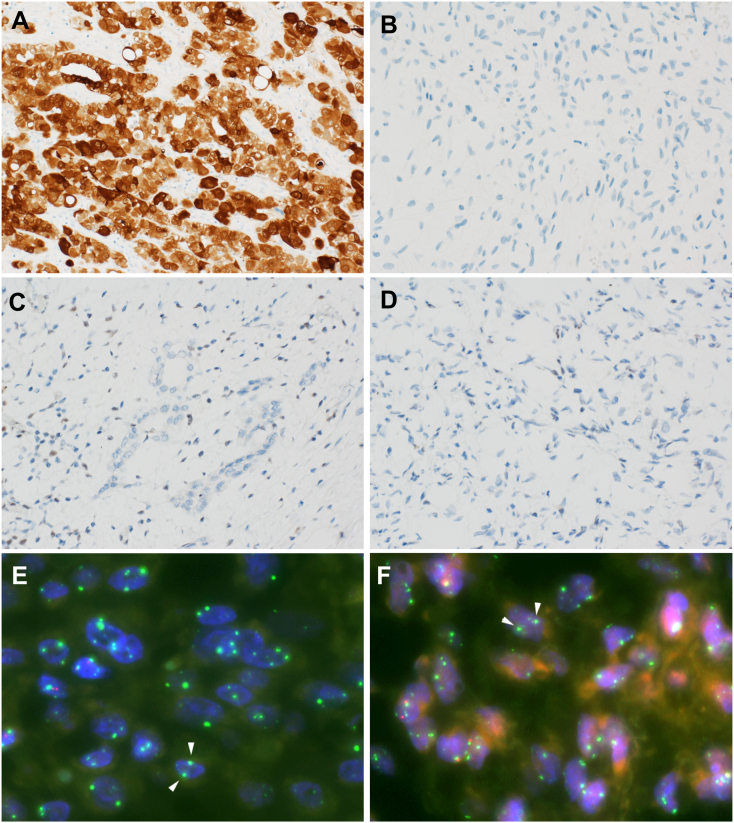
Immunohistochemistry and fluorescence *in situ* hybridization (FISH). Immunohistochemically, epithelial cells were positive for calretinin (A). Spindle-shaped cells were negative for calretinin (B). The loss of expression of BAP-1 was identified in both the epithelial (C) and spindle-shaped cells (D). FISH showed homozygous deletions of *CDKN2A* in both epithelial (E) and spindle-shaped cells (F). Green (arrows) and red signals indicated 9p centromere and *CDKN2A*, respectively.

**Fig. 3:**
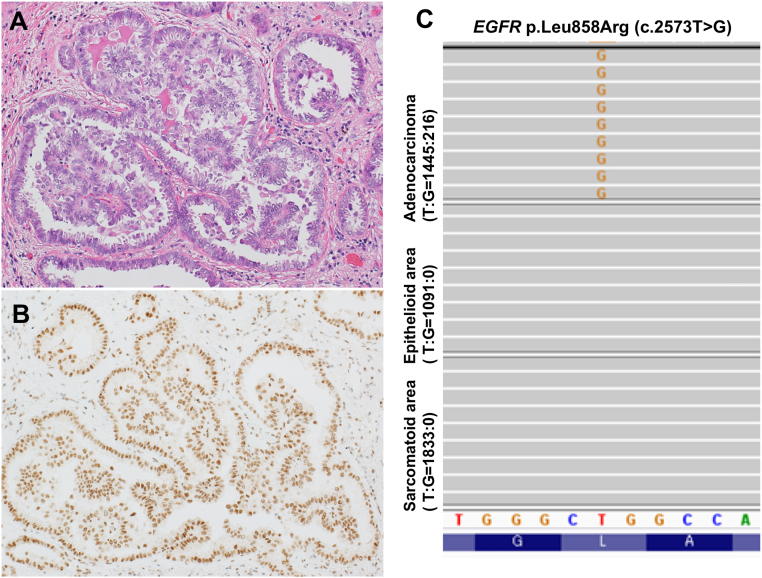
Pathological findings of lung adenocarcinoma. (A) Tumor cells displaying papillary architecture. (B) Immunohistochemically, BAP1 expression was retained. (C) Mutation of *EGFR* p.Leu858Arg (c.2573T>G) were detected.

In immunohistochemical analysis, the epithelial cells were observed to be diffusely and strongly positive for CAM5.2, AE1/AE3, calretinin, mesothelin, D2–40, and HEG-1, whereas spindle-shaped cells were negative for all of them, except for D2–40 (Fig. 2A, B). Both epithelial and spindle-shaped cells were negative for WT-1, CEA, Ber-EP4, and TTF-1. The loss of BAP1 (BRCA1-associated protein 1) expression in both the components was observed (Fig. 2C, D). Additionally, fluorescence *in situ* hybridization (FISH) revealed homozygous deletions of *CDKN2A* in both the components (Fig. 2E, F). RT-PCR analysis revealed no alternative splice forms of *SS18*-*SSX* fusion, which led to the exclusion of synovial sarcoma (data not shown). Based on histological, immunohistochemical, and molecular analyses, we made a diagnosis of diffuse pleural mesothelioma, the biphasic subtype. In contrast, the nodule in the lung parenchyma was papillary adenocarcinoma that was immunohistochemically negative for all mesothelial markers, including calretinin, and retained BAP1 expression (Fig. 3A, B). To further unravel such an unusual case in which lung adenocarcinoma and malignant mesothelioma were simultaneously identified, next-generation sequencing was performed using the 50-gene Ion AmpliSeq Cancer Hot Panel v2 approach with the Ion GeneStudio S5 (Thermo Fisher Scientific, Waltham, MA, USA). Mutations in *EGFR* (L858R) were documented with 13% allelic frequency in lung adenocarcinoma (Fig. 3C), whereas no mutations were detected in both the components of biphasic mesothelioma, although it was unclear whether tumor cells harbored *BAP1* mutations because Ion AmpliSeq Cancer Hot Panel v2 does not include *BAP1*. The patient is alive and well, with no evidence of disease recurrence 7 months after surgery.

## 3 Discussion

Here, we described a patient with biphasic mesothelioma of the pleura showing rare morphology mimicking the myxofibrosarcoma concomitant with *EGFR*-mutated lung adenocarcinoma. In the present case, the pleural tumor macroscopically showed a myxoid appearance and microscopically exhibited epithelioid and sarcomatous components that were divided by a fibrous septum, leading us to consider the possibility of epithelioid mesothelioma concurrent with myxofibrosarcoma, although such combinations are extremely rare. Generally, the immunoreactivity for mesothelial markers including calretinin, CK5/6, WT1, and D2–40 is helpful for distinguishing malignant mesothelioma from other malignant tumors proliferating in the pleura. However, the role of immunohistochemistry is more limited for sarcomatoid mesothelioma than for epithelioid mesothelioma because staining for mesothelial markers in epithelioid mesothelioma is often less positive than that in sarcomatoid mesotheliomas [1]. In such cases, similar to the present case, ancillary studies for *CDKN2A* deletion or BAP1 immunohistochemistry can be helpful [6], [7], [8]. An important consideration for the differential diagnosis of biphasic mesothelioma with myxoid stroma includes other biphasic tumors, such as carcinosarcoma and synovial sarcoma. Although no *SS18*-*SSX* fusion led us clearly exclude synovial sarcoma in the present case, the histologic distinction of synovial sarcoma from malignant mesothelioma may be difficult because of the combination of epithelioid and spindle cells, potentially sharing locations, and immunopositivity for calretinin in both tumors [9].

Synchronous presentation of lung adenocarcinoma and malignant mesothelioma is uncommon. Such rare combinations and absence of asbestos exposure in the present case raised the possibility of the tumor being associated with *BAP1* germline alterations (BAP1 tumor predisposition syndrome) [10]. Indeed, there is a documented case of germline *BAP1* mutation with multiple primary cancers, including synchronous lung adenocarcinoma with concurrent *BAP1* and *ERBB2* mutations, bilateral malignant pleural mesothelioma, metachronous peritoneal malignant mesothelioma, intrahepatic cholangiocarcinoma, bladder urothelial carcinoma, and prostatic adenocarcinoma [11]. However, unlike that case, our patient had no family history of cancer, malignant tumors other than lung adenocarcinoma and mesothelioma, and loss of BAP1 expression in lung adenocarcinoma. Although we were unable to determine whether the patient harbored a germline *BAP1* mutation, screening for germline *BAP1* status might be needed.

Our case was unique because the tumor areas mimicked the myxofibrosarcoma, which contains myxoid stroma. Epithelioid mesothelioma with a pronounced myxoid stroma has been identified as a morphological pattern that may lead to a better prognosis [12]. Although alcian blue stained mucins were detected in subsets of biphasic mesothelioma [13], the clinicopathological significance of myxoid stroma in biphasic and sarcomatoid mesothelioma remains largely unknown, and requires further investigation.

## 4 Conclusion

Our case presented a unique morphology mimicking the myxofibrosarcoma in a sarcomatoid component of biphasic mesothelioma; therefore, it raises a question of the clinicopathological significance of myxoid stroma in sarcomatous areas of biphasic and sarcomatoid mesothelioma.

## Ethical approval

Written informed consent was obtained from the patient. The project was ethically approved by the institutional review board of Juntendo University (JH21-014).

## Funding information

This study was supported in part through grants from the Grant-in-Aid for Scientific Research (KAKENHI) program of the 10.13039/501100001691Japan Society for the Promotion of Science (JSPS) (Grant Number #19K07469).

## Author contributions

Takuo Hayashi and Tsuyoshi Saito conceived and designed the study. Hiroko Onagi and Takuo Hayashi wrote, edited, and reviewed the manuscript. Kazuya Takamochi and Kenji Suzuki provided the patient with clinical information. Hiroko Onagi, Takuo Hayashi, Tsuyoshi Saito, and Satsuki Kishikawa provided the pathological information. All authors have approved the final manuscript for publication. Takuo Hayashi takes full responsibility for the work as a whole, including the study design, access to data, and the decision to submit and publish the manuscript.

## Guarantor

Takuo Hayashi.

## Research registration number

Not applicable.

## Data availability statement

All data generated or analyzed during this study are included in this article. Further enquiries can be directed to the corresponding author.

## Provenance and peer review

Not commissioned, externally peer-reviewed.

## Declaration of competing interest

None declared.
